# Separation of trait and state in stuttering

**DOI:** 10.1002/hbm.24063

**Published:** 2018-04-06

**Authors:** Emily L Connally, David Ward, Christos Pliatsikas, Sarah Finnegan, Mark Jenkinson, Rowan Boyles, Kate E Watkins

**Affiliations:** ^1^ Wellcome Centre for Integrative Neuroimaging, University of Oxford Oxford United Kingdom; ^2^ Department of Experimental Psychology University of Oxford Oxford United Kingdom; ^3^ Nuffield Department of Clinical Neurosciences , University of Oxford Oxford United Kingdom; ^4^ School of Psychology and Clinical Language Sciences University of Reading, Reading United Kingdom

**Keywords:** basal ganglia, cerebellum, developmental stuttering, movement disorder, speech disorder

## Abstract

Stuttering is a disorder in which the smooth flow of speech is interrupted. People who stutter show structural and functional abnormalities in the speech and motor system. It is unclear whether functional differences reflect general *traits* of the disorder or are specifically related to the dysfluent speech *state*. We used a hierarchical approach to separate state and trait effects within stuttering. We collected sparse‐sampled functional MRI during two overt speech tasks (sentence reading and picture description) in 17 people who stutter and 16 fluent controls. Separate analyses identified indicators of: (1) general ***traits*** of people who stutter; (2) frequency of dysfluent speech states in subgroups of people who stutter; and (3) the differences between fluent and dysfluent ***states*** in people who stutter. We found that reduced activation of left auditory cortex, inferior frontal cortex bilaterally, and medial cerebellum were general traits that distinguished fluent speech in people who stutter from that of controls. The stuttering subgroup with higher frequency of dysfluent states during scanning (*n* = 9) had reduced activation in the right subcortical grey matter, left temporo‐occipital cortex, the cingulate cortex, and medial parieto‐occipital cortex relative to the subgroup who were more fluent (*n* = 8). Finally, during dysfluent states relative to fluent ones, there was greater activation of inferior frontal and premotor cortex extending into the frontal operculum, bilaterally. The above differences were seen across both tasks. Subcortical state effects differed according to the task. Overall, our data emphasise the independence of trait and state effects in stuttering.

## 1 INTRODUCTION

Persistent developmental stuttering is a disorder marked by disruptions to the smooth flow of speech that emerges in early childhood. By adulthood, roughly 1 in 100 individuals continue to stutter (Felsenfeld, 2002). All of these individuals have periods of fluent speech as well as the periods of dysfluent speech that define their disorder. *State effects* distinguish specific states of dysfluent from fluent speech. Specific states of dysfluent speech typically include core characteristics of stuttering, for example, repetitions, prolongations, and blocking of sounds, which most frequently occur at the beginnings of words. The distinction between states is obvious to most listeners, and many people who stutter can predict specific situations and sounds that are likely to result in dysfluent states. Still, the field of stuttering research has yet to isolate the neural correlates of state effects in stuttering. Research investigating trait effects in stuttering, that is, what distinguishes people who stutter from people who are fluent, is abundant, but some major areas of disagreement still exist. Distinguishing between brain activity that reflects *traits* of stuttering, that is, “commonalities among people who stutter”; and *states* of stuttering, that is, “disruptions that are associated with the act of stuttering” is critical in unravelling the underpinnings of this developmental disorder of speech fluency and resolving discrepancies in the literature.

### 1.1 Speech‐motor system traits in stuttering

Disorganisation of white matter is a structural trait of developmental stuttering. Microstructural disruption was first thought to be localized to a critical speech‐motor pathway underlying the left central opercular cortex (Sommer, Koch, Paulus, Weiller, & Büchel, 2002). These disruptions to left hemisphere white matter microstructure underlying key motor areas have also been documented during middle childhood in children who continue to stutter, as well as in those who had recovered (Chang, Erickson, Ambrose, Hasegawa‐Johnson, & Ludlow, 2008). Further studies reported that the disorganisation extended into ventral premotor cortex, bilaterally, and corresponded to abnormalities in functional activation of nearby grey matter in adolescents and young adults who stutter (Watkins, Smith, Davis, & Howell, 2008). In adults who stutter, the right hemisphere disruptions underlying ventral premotor tracts correspond to hyperactivity during imagined speech tasks in fMRI and correlate with stuttering severity (Neef et al., 2017).

Previous work suggests differences follow a more diffuse pattern of white matter disorganisation, with many disruptions occurring bilaterally (Connally, Ward, Howell, & Watkins, 2014). White matter was disorganized in each of the four cerebral lobes, in the cerebral and cerebellar peduncles, and within the posterior body of the corpus callosum. Notably, descending motor pathways (corticobulbar tracts) showed greater abnormality in the left hemisphere compared to the right within people who stutter, whereas fluent controls showed no differences between hemispheres in these pathways. Overall, abnormalities have been documented in numerous white matter pathways in the brains of individuals who stutter, and differences are especially pronounced in the left hemisphere speech‐motor pathways (Neef, Anwander, & Friederici, 2015). It remains to be determined whether these tracts are affected early or even before the onset of the disorder in early childhood. Given that children who stutter show predominately left‐hemisphere disorganization (Chang et al., 2008), it is likely that some, if not all, of the microstructural differences observed in adults who stutter relate to experience‐dependent mechanisms that surface throughout the duration of the disorder.

Functional neuroimaging studies of stuttering also show diffuse disruption in speech‐motor networks. A seminal meta‐analysis summarized findings as three “neural signatures of stuttering”: overactivity in the right inferior frontal cortex and anterior insula; underactivity in the auditory cortex; and overactivity in the cerebellar vermis (Brown, Ingham, Ingham, Laird, & Fox, 2005). These traits emerged in spite of variability of neuroimaging method, tasks used, and fluency state. Two more recent meta‐analyses replicated in part the originally‐reported neural signatures and further attempted to dissociate state from trait effects (Belyk, Kraft, & Brown, 2017; Budde, Barron, & Fox, 2014). One meta‐analysis reported stuttering trait effects in terms of overactivity of right ventral premotor/motor cortex and Rolandic operculum, and underactivity of left ventral premotor/motor cortex and found that the right ventral motor cortex overactivity (at the level of the face representation) was specific to the stuttering state (Belyk et al., 2017). The other meta‐analysis (Budde et al., 2014) reclassified reduced auditory cortex activity to be both trait (on the left) and state (bilaterally but predominantly on the right) effects and cerebellar overactivity as a state effect; overactivity of the right inferior frontal areas including the anterior insula were classified as definite trait and possible state effects. In addition, this meta‐analysis found that overactivity of the supplementary motor complex (SMA and preSMA) was a general trait that distinguishes individuals who stutter from fluent controls as well as a state effect, as it was seen during dysfluency (Budde et al., 2014).

### 1.2 Attempts to distinguish state effects in stuttering

Direct comparisons of dysfluent to fluent speech states within individuals are rare. Of the four reports to do so (den Ouden, Montgomery, & Adams, 2014; Jiang, Lu, Peng, Zhu, & Howell, 2012; Sowman, Crain, Harrison, & Johnson, 2012; Wymbs, Ingham, Ingham, Paolini, & Grafton, 2013) both fluent and dysfluent speech states recruited the same fundamental network, which is consistent with findings in studies of trait effects indicating similarities in activation are also far greater than differences (Fox et al., 1996; Ingham, Grafton, Bothe, & Ingham, 2012). Specific findings can loosely be grouped according to those showing reduced recruitment of language‐dominant inferior frontal cortex during dysfluent speech (den Ouden et al., 2014; Sowman et al., 2012), those studies showing increased recruitment of the speech‐motor network during dysfluent speech (Sowman et al., 2012; Wymbs et al., 2013), and a single study reporting both these patterns of alterations and suggesting they underlie different specific speech symptoms in stuttering (Jiang et al., 2012). Within participants, both the basal ganglia and cerebellum are associated with lower magnitude of signal change during “more typical” relative to “less typical” stuttering symptoms (Jiang et al., 2012). In other words, the “less typical” symptoms of stuttering (incomplete phrases, revisions, interjections, and phrase repetitions) resulted in subcortical overactivity relative to “more typical” symptoms (part‐word repetitions, prolongations, broken words) (Jiang et al., 2012). The distinction between specific symptoms within dysfluent speech states suggests potential for dual‐roles of the subcortical motor structures in stuttering neuropathology. Further, the variability in the specific location of state effects cannot be overstated, as only a single region overlapped across all individuals in one study: the left lobule IV of the cerebellum was overactive for dysfluent relative to fluent speech in four adults males who stutter in whom different speech states were directly contrasted (Wymbs et al., 2013).

To a certain extent, relationships between neural activation and stuttering severity or treatment gains can supplement the dearth of direct examinations of dysfluent speech. Application of such efforts is often limited due to choice of measurement. For example, in some studies, “stuttering severity” was actually a measure of frequency of dysfluent states (e.g., Giraud et al., 2008), whereas in other studies a standardized instrument was used that calculated a composite score using stuttering frequency, duration of stuttered events, and subjective severity of concomitant behaviors (Stuttering Severity Instrument, SSI [Riley, 1994], e.g., Sakai, Masuda, Shimotomai, & Mori, 2009). One common factor in reports of the neural correlates of stuttering severity is a proclivity to more frequent dysfluency (Belyk et al., 2017), which may involve different circuits to those underlying the presence or absence of dysfluent speech, *per se*. Still, abnormal activity in subcortical structures is frequently implicated in relation to stuttering severity: cerebellar activity normalizes following treatment (De Nil, Kroll, & Houle, 2001; Lu, Arai, Tsai, & Ziemann, 2012), as does basal ganglia activity (Toyomura, Fujii, & Kuriki, 2015). Speech‐related activation in the basal ganglia was positively correlated with stuttering frequency using both PET (Ingham et al., 2012) and fMRI (Giraud et al., 2008). Generally, many studies implicate some portion of the basal ganglia or cerebellum or both in stuttering states, but the precise roles of these structures are little understood.

### 1.3 The aims of the current study

**Figure 1:**
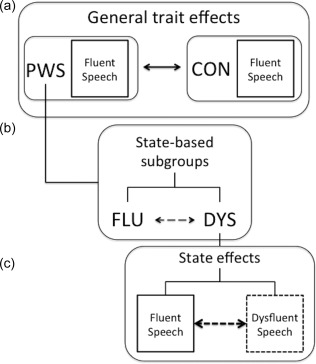
Isolating trait and state effects in stuttering. (a) We conceptualised general traits as those things shared by people who stutter (PWS) that are different when compared to fluent controls (CON). In this way, activity during fluent speech, which occurs in both PWS and CON, can be used to isolate a general trait of stuttering. (b) In our study, about half of the PWS group overlapped with CON in the average number of dysfluent utterances per scan; the other half did not overlap with CON at all in terms of frequency of dysfluent utterances. Therefore, we used a data–driven threshold to create two subgroups of PWS: mostly fluent (FLU) and somewhat dysfluent (DYS). These subgroups were compared directly to isolate effects related to a general “proclivity” to dysfluency in the scanning environment. (c) Within the DYS subgroup, we were able to isolate specific state effects through comparing dysfluent to fluent speech epochs directly

Here, we used a whole‐brain approach to explore speech‐related activity during two speech production tasks—sentence reading and picture description—as reflecting either general traits of stuttering or specific states of stuttered speech. We defined trait and state effects in stuttering as follows (Figure 1):

- General Traits: Activity during fluent‐speech production in people who stutter (PWS) that differed from that in controls (CON).
- State‐based subgroups: We divided our sample of PWS, who all share the stuttering trait, into two subgroups using a data‐driven threshold for fluency during scanning: one subgroup contained individuals who were mostly fluent (FLU) during scanning and the other contained participants who were more dysfluent (DYS).
- State effects: Within individuals in the DYS subgroup, we compared fluent to dysfluent utterances to explore potential indicators of the stuttering state.

## 2 MATERIALS AND METHODS

### 2.1 Participants

Seventeen adults with persistent developmental stuttering (PWS: 13 males, 4 females; aged 19–54 years; 3 left‐handers) and 17 age and sex‐matched fluent controls (CON: 13 males, 4 females; aged 19–53 years; 3 left‐handers) were scanned using functional MRI. All participants were native English speakers. No controls had a history or diagnosis of learning or speech disorders. All participants gave informed consent to their participation in the research in a protocol approved by University of Reading's ethics committee. We removed one male control participant from this analysis because of noncompliance with instructions (see “Speech tasks” below for details). Therefore, a total of 16 CON and 17 PWS were retained for analysis.

Groups (CON and PWS) were well‐matched on age, education, handedness and gender (Table 1). This work is part of a larger study on fluency disorders, therefore data from the CON group reported here was also used as in a previously published report of fMRI activity in a different speech disorder known as cluttering (Ward, Connally, Pliatsikas, Bretherton‐Furness, & Watkins, 2015). That previous report did not include these PWS data nor focus on fluent speech within the CON group as we have in the current study.

**Table 1:** Summary of group and subgroup demographics

				PWS Subgroups	
		CON	PWS	FLU	DYS
Total n	[females]	16[4]	17[4]	8[3]	9[1]
Left handed	[females]	2[0]	3[1]	2[1]	1[0]
Age in years	Mean (SD)	33.1 (11.4)	31.4 (11.0)	31.75 (14.4)	31.11 (7.8)
	Range	19–53	19–54	22–45	19–54
Education in years	Mean (SD)	17.8 (1.9)	16.35 (1.8)	16.25 (0.7)	16.44 (1.9)
Stuttering severity	Median		23	19	30
	Range		10–46	10–43	10–46

Stuttering ranged in severity from very mild to very severe, as assessed using the Stuttering Severity Instrument (SSI–III, [Riley, 1994] median 23, interquartile range 16.5–32.5, range 10–46). Most of the participants in the PWS group had received treatment previously, which varied in terms of type, ages at which received, and the interval relative to the time of study involvement. Importantly, none were receiving treatment at the time of the study, nor had they been involved in a treatment program for at least one year prior to participation.

**Figure 2:**
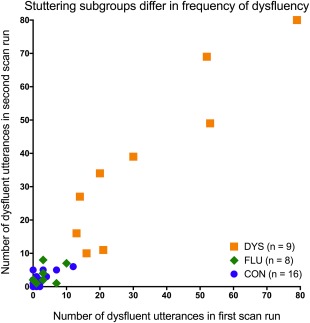
Stuttering subgroups differed in terms of the frequency of dysfluent utterances during scan runs. This plot reflects how we determined subgroups of people who stutter using a data‐driven threshold of 10 dysfluent utterances per scan run. The mostly fluent (FLU) subgroup overlapped entirely with the fluent control group (CON) in their frequencies of dysfluent speech in the scanner. The somewhat dysfluent stuttering subgroup (DYS) did not overlap with either CON or FLU in terms of the number of dysfluent utterances across scan runs [Color figure can be viewed at http://wileyonlinelibrary.com]

We separated our PWS group into two state‐based subgroups (FLU and DYS), which differed in the frequency of dysfluent speech during scanning (Figure 2). The threshold for subgrouping meant that participants in the DYS group needed to have at least ten dysfluent utterances in each scan run. The distribution of frequencies of dysfluent utterances of the FLU subgroup overlapped entirely with that of the CON group. The DYS and FLU subgroups were not overlapping in terms of numbers of dysfluent utterances (Figure 2). However, the subgroups were overlapping in terms of the range of their stuttering severity scores, and did not significantly differ in stuttering severity (SSI–III total score), age, or years of education (Table 1).

### 2.2 Data acquisition

Functional MRI data were obtained at the University of Reading's Centre for Integrative Neuroscience and Neurodynamics using a 3T Siemens Trio scanner with a 12‐channel head coil. Whole head T2*‐weighted echo planar images (TE = 30 ms) were acquired every 9 s with a silent delay of 7 s (i.e., sparse sampling) and comprised 2‐s acquisition of 32 4‐mm axial slices (in plane resolution 3 × 3 mm). A “+” appeared in the middle of the screen during the 2‐s acquisition period. During the 7‐s silent delay between measurements, participants saw a stimulus via scanner compatible goggles that was a picture with a descriptive sentence below it, or a picture with no text, or a “+” in the middle of the screen.

Participants were instructed to read the sentences aloud (Sentence Reading task) or to overtly describe the pictures (Picture Description task) and were explicitly told to stop speaking when the crosshair appeared so that there would be no speech related movement of the head during data collection. Prior to the scan, the task was explained to participants, who were allowed to practise outside the scanner. Participants were explicitly instructed to refrain from using fluency enhancement techniques in the scanner and to speak as naturally as possible. For each of the tasks and the baseline, 40 volumes of data were acquired for a total of 120 volumes (18 min); the order of tasks was fixed and pseudorandom. Two runs were acquired in each participant, yielding a total of 80 volumes of each task (Sentence Reading, Picture Description) and baseline.

### 2.3 Speech tasks

We use two tasks to elicit overt speech: (1) sentence reading and (2) picture description. These tasks were described previously in a report of fMRI activity in cluttering, a disorder which shares some symptoms and is often comorbid with stuttering (Ward et al., 2015). Speech was recorded using an MRI‐compatible microphone. A native British‐speaker (S.F.) was blinded to participant group and asked to rate recorded utterances as either dysfluent or fluent. The rater was given instructions to mark utterances as either fluent (0) or dysfluent (1: “if speech is dysfluent at all”). These instructions were followed by guidelines summarising the symptoms of two fluency disorders: stuttering (American Psychiatric Association, 2013) and cluttering (Louis & Schulte, 2011). A sentence was considered dysfluent if it contained at least one interruption to speech flow. Such dysfluencies included core stuttering characteristics (repetition, prolongation or tense pauses/blocks) as well as other speech dysfluencies, such as repeated multisyllabic words or phrases, revisions, fragments, or interjections (e.g., “um”). It is worth noting that the latter occur in the speech of normally fluent speakers as well as in the speech of people who stutter, but do not contribute towards calculations of stuttered syllables in the SSI.

The blinded ratings were compared to ratings from another native British‐speaker (R.B.) who was provided with the same rubric but was not blind to participant group. Item by item agreement was calculated between the two raters. The resulting agreement was high (0.93) across all items (160 utterances) and all participants in this study (*n *=* *34*)*, as well as an additional sample with a fluency disorder known as cluttering (*n *=* *17).

We were able to directly confirm participant compliance with task instructions via the recordings we took in the scanner. During this process we noticed three types of speech errors: (1) describing the picture instead of reading a sentence; (2) continuing to speak after reading the sentence; and (3) speaking during baseline trials. When participants made the first type of error we re‐coded the trial as a “picture description” trial rather than a sentence reading trial. This was the case for eight trials across all participants (one for each of 1 CON and 2 PWS, and five trials in one other PWS). For the other types of errors, when possible we removed trials using a confound variable for each trial, as is done for motion outliers in FSL. This process was used to remove 1 sentence reading trial in a CON who added speech, 3 baseline trials in one PWS, and 10 baseline trials in another PWS. For two CON participants, the errors were too frequent to be addressed in this fashion. Therefore, one control participant was removed from the study for continuing to speak after finishing reading on 69/80 “sentence reading” trials. One entire session from another control participant was removed from the study for making the same error on 38/40 sentence reading trials in the first session. The second scan session was retained for this participant.

### 2.4 Image analysis

#### 2.4.1 Preprocessing

The functional images were analysed using the FMRIB Software Library (FSL 5.0.6; http://www.fmrib.ox.ac.uk/fsl, [Jenkinson, Beckmann, Behrens, Woolrich, & Smith, 2012]). Standard motion correction, and individual volumes that were motion outliers were included as separate regressors at the first level for each participant. Excessive motion, defined as larger than the widest voxel dimension, (i.e., >4 mm) was observed towards the end of a single scan run in one PWS and one CON, and these volumes were removed from the time series (i.e., the runs were truncated by 29 volumes (PWS) and 20 volumes (CON) within a single 120 volume run).

The remaining data were analysed in the same way for all participants. Each dataset was unwarped using a fieldmap and PRELUDE and FUGUE software running in FSL (Jenkinson et al., 2012) and spatially smoothed with an 8‐mm full‐width‐at‐half‐maximum smoothing kernel. A temporal high‐pass filter with a cut‐off of 150 s was used to remove low‐frequency fluctuations in the signal. Two further regressors were used in the first‐level analysis to remove residual image artefacts due to physiological changes. These regressors were the mean time‐courses extracted from preprocessed data from 4‐mm radius spheres in areas where task‐related activity was not expected; one was placed within cerebrospinal fluid of the anterior lateral ventricle (standard space coordinates 2, 10, 8) and the other within white matter in the dorsal posterior frontal lobe (–26, −22, 28) (Leech, Braga, & Sharp, 2012). Images were registered using boundary‐based registration (Greve & Fischl, 2009) to the individual participant's T1‐weighted structural image (1 mm^3^ voxels; TR = 2,020 ms, TE = 2.9 ms, flip angle = 90°), which in turn was registered using FNIRT (FMRIB's nonlinear registration tool) to the MNI‐152 template.

#### 2.4.2 Modelling of trait and state effects

*General traits*: We defined stuttering traits as abnormal activity observed during fluent speech in PWS relative to that observed in CON (Figure 1). For individual participants, for each scan run, statistical maps were generated to show patterns of activation during each task relative to baseline. Each task was modelled using a separate regressor. To examine these trait‐based effects, for all participants (including CON), we added a behavioural regressor at the first‐level coding for dysfluent utterances. This regressor removed the residual effect of dysfluent utterances for each scanner run, thereby allowing us to restrict our analyses to fluent speech.

At the first level, we modelled five contrasts of interest for each participant: the average for each task relative to baseline, the contrast of each task to the other (in both directions), and the average of all speech (both tasks) relative to baseline, for use in higher level models testing a group factor. In a second‐level analysis, to combine data from the two runs, we averaged statistical maps from the first level for each participant using a fixed‐effects analysis.

At the highest level, a 2 (task) × 2 (group) ANOVA model was implemented using FMRIB's Local Analysis of Mixed Effects stage 1 (Woolrich, Behrens, Beckmann, Jenkinson, & Smith, 2004). We probed for the main effect of task (sentence reading and picture description) and the interaction between task and group (PWS and CON) in a single model. Main effects of group were probed using unpaired *t* tests on the average signal of “all speech” relative to baseline across both tasks.

*State‐based subgroups*: In a second analysis, we divided our PWS sample into subgroups of individuals who were mostly fluent (FLU, *n *=* *8) or dysfluent (DYS, *n *=* *9) based on a cut‐off of at least 10 dysfluent utterances per scan run. The cut‐off was based on the number of utterances that were dysfluent in both scans (Figure 2). The mostly fluent subgroup (FLU) completely overlapped with the CON group in terms of the frequency of dysfluent speech in the scanner. The other subgroup, DYS, did not overlap with either FLU or CON. In other words, we set a cut‐off independent of speaking task, yet still indicative of general proclivity to stuttering throughout the scan session. For this analysis, we included both fluent and dysfluent speech states, and therefore did not use an additional regressor for dysfluent utterances. Otherwise all aspects of analyses were identical to the general trait pipeline described above, though at the highest level we compared FLU and DYS subgroups rather than PWS and CON (Figure 1).

*State effects*: Within the DYS subgroup, we explored activity related to the two types of speech states: fluent and dysfluent (Figure 1). We set a minimum of 10 fluent or 10 dysfluent utterances per run for inclusion in this analysis. Therefore, one DYS participant who had only a single completely fluent utterance across both runs, was excluded from this analysis. For another DYS participant, we excluded one run, which contained only a single dysfluent “sentence reading” trial but 15 dysfluent “picture description” trials, resulting in an imbalance in ANOVA cells. We refer to this analysis as comparing dysfluent and fluent states, which are approximated by comparison of dysfluent to fluent utterances.

In this analysis, we were primarily interested in (1) isolating state effects; and (2) detecting interactions between state and task. We coded four variables at the first level so that we could examine activity related to fluent and dysfluent states within the Picture Description and Sentence Reading tasks, respectively. At the second level, as described above, we averaged the two scanner runs for each participant, with the exception of one participant for whom data were only available from one run (see above).

Finally, at the highest level, we implemented, a 2 (task) × 2 (state) within ANOVA model using FMRIB's Local Analysis of Mixed Effects stage 1 (Woolrich et al., 2004). We probed for the main effect of task (Sentence Reading and Picture Description), the main effect of state (fluent and dysfluent) and the interaction between them in a single model. Significant effects were explored using *t* tests that averaged effects across the group.

*Motion effects*: The dysfluent state can be accompanied by physical concomitants (jerks, tics, and other orofacial movements). This could result in greater movement during scans acquired after trials containing dysfluency and consequently affect the signal in those images. To address this concern, we directly tested the hypothesis that movement was greater during dysfluent trials than fluent trials. We computed an average of the absolute values for each of the six standard motion correction parameters for fluent and dysfluent items within each scan run for individuals whose data were used in the state analysis. We then used a 2 × 6 repeated measures ANOVA to probe for excess movement‐related noise in dysfluent relative to fluent states. The motion parameters did not differ significantly between states, nor did they interact with state.

We then examined whether there was a difference in the proportion of motion outliers detected during each speech state. We first calculated the proportion of the total number of motion outliers to the total number of items for each speech state for each participant in the state analysis. We then used a paired *t* test to compare states within participants. The proportion of items detected as motion outliers did not differ significantly between states. We were able, therefore, to reject the hypothesis that images acquired after trials containing dysfluent speech required greater motion correction than did images acquired after trials containing fluent speech.

*Thresholding*. The highest level of each analysis (for group, subgroup or state, respectively) was examined first using a cluster forming threshold of *Z *>* *2.3 and a cluster significance threshold of *p < *.05, corrected for multiple comparisons. When the modelled main effects or interactions did not reveal any significant differences at this corrected threshold, we used an exploratory, uncorrected threshold of *p* < .01 (*Z *>* *2.3), with an additional constraint that the cluster size was at least 30 voxels, located primarily in grey matter.

## 3 RESULTS

### 3.1 Behavioural results

Table 2 summarises the data for dysfluent utterances during scanning in each group and subgroup. We conducted Kruskal‐Wallis tests to compare frequency of dysfluent utterances during scanning between our groups (PWS and CON) and subgroups (FLU and DYS). PWS had significantly more dysfluent utterances than CON in the sentence reading task (*p* = .003), but this difference did not reach significance in the picture description task (*p* = .058). As expected (because subgroups were selected according to frequency of dysfluency across scans), the DYS subgroup was significantly less fluent than the FLU subgroup in both tasks (sentence reading [*p* = .029] and picture description [*p* = .014]). The FLU subgroup did not differ from CON in either task. Further, there were no significant differences between tasks in terms of the frequency of dysfluent utterances in sentence reading relative to picture description within any group nor in the PWS subgroups (Table 2).

**Table 2:** Dysfluency during scanning. Mean and standard deviations for number of dysfluent sentences during the tasks for PWS, CON, and the 2 subgroups of PWS: FLU and DYS

Participants	Task	Mean (*SD*)	Min	Max
*CON* (*n* = 16)				
	Sentence reading	1.59 (1.9)	0	6
	Picture description	3.18 (3.6)	0	12
*PWS* (*n* = 17)				
	Sentence reading	20.18 (23.7)	0	80
	Picture description	20.24 (23.9)	1	79
*FLU* (*n* = 8)				
	Sentence reading	4.25 (4.7)	0	14
	Picture description	2.50 (1.2)	1	5
*DYS* (*n* = 9)				
	Sentence reading	34.3 (25.0)	4	80
	Picture description	36.0 (23.5)	9	79

Minimum and maximum counts are summed across both scan runs.

### 3.2 Neuroimaging results: General traits

The trait analysis compared activity in the PWS and CON groups during fluent utterances spoken either during picture description or the sentence reading tasks. The analysis revealed significant task differences (cluster forming threshold *Z *>* *2.3, family wise error corrected to *p < *.05*)* across both groups. Group differences across task and interactions between trait and task were not significant at the corrected threshold, so we present exploratory trait effects observed in grey matter voxels at a more lenient threshold (*p* < .01 and extent *k *≥* *30 voxels).

#### 3.2.1 Task effects

**Figure 3:**
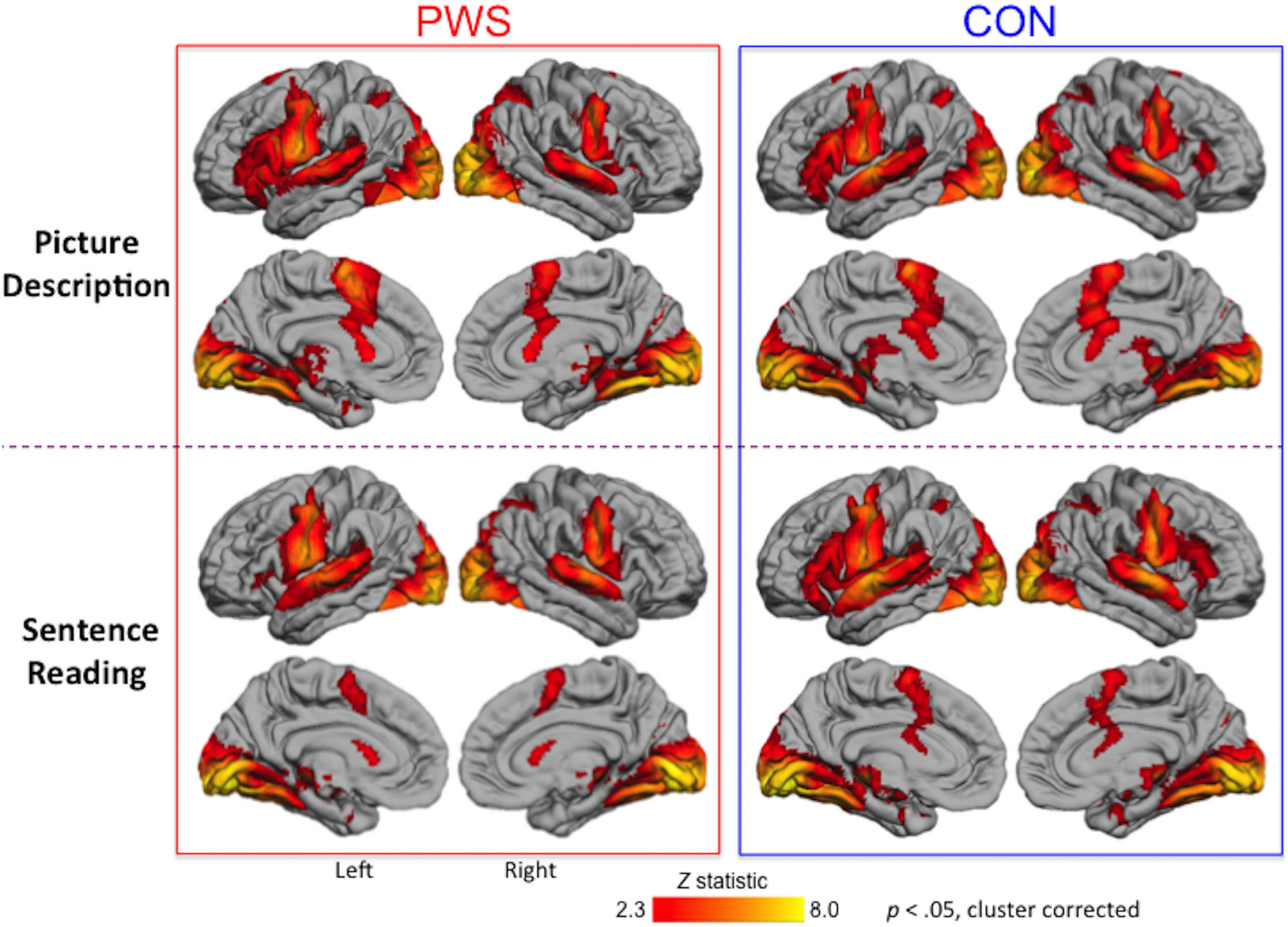
Fluent speech‐related activity: People who stutter (PWS), and fluent controls (CON) activated the same network during picture description (top two rows) and sentence reading (bottom two rows). Coloured statistical maps were thresholded (*Z* > 2.3, *p* < .05) and superimposed on the lateral and medial surfaces of the left and right hemisphere using FreeSurfer [Color figure can be viewed at http://wileyonlinelibrary.com]

The difference between the picture description and sentence reading tasks across both groups was not of primary interest in this study other than in the case where it differed between groups (i.e., task by group interaction). Therefore, we present the results of the task analysis briefly here and in further detail in supplementary material. In both tasks, there was activation across the expected network of areas bilaterally, namely posterior superior temporal cortex, sensorimotor cortex, SMA and preSMA, and the left posterior inferior frontal gyrus (IFG). In addition, there was extensive medial and lateral occipital cortex activity (see Figure 3). As expected there was significantly more activity throughout this network for the picture description task relative to sentence reading (for details see supplementary material).

#### 3.2.2 Trait effects

**Figure 4:**
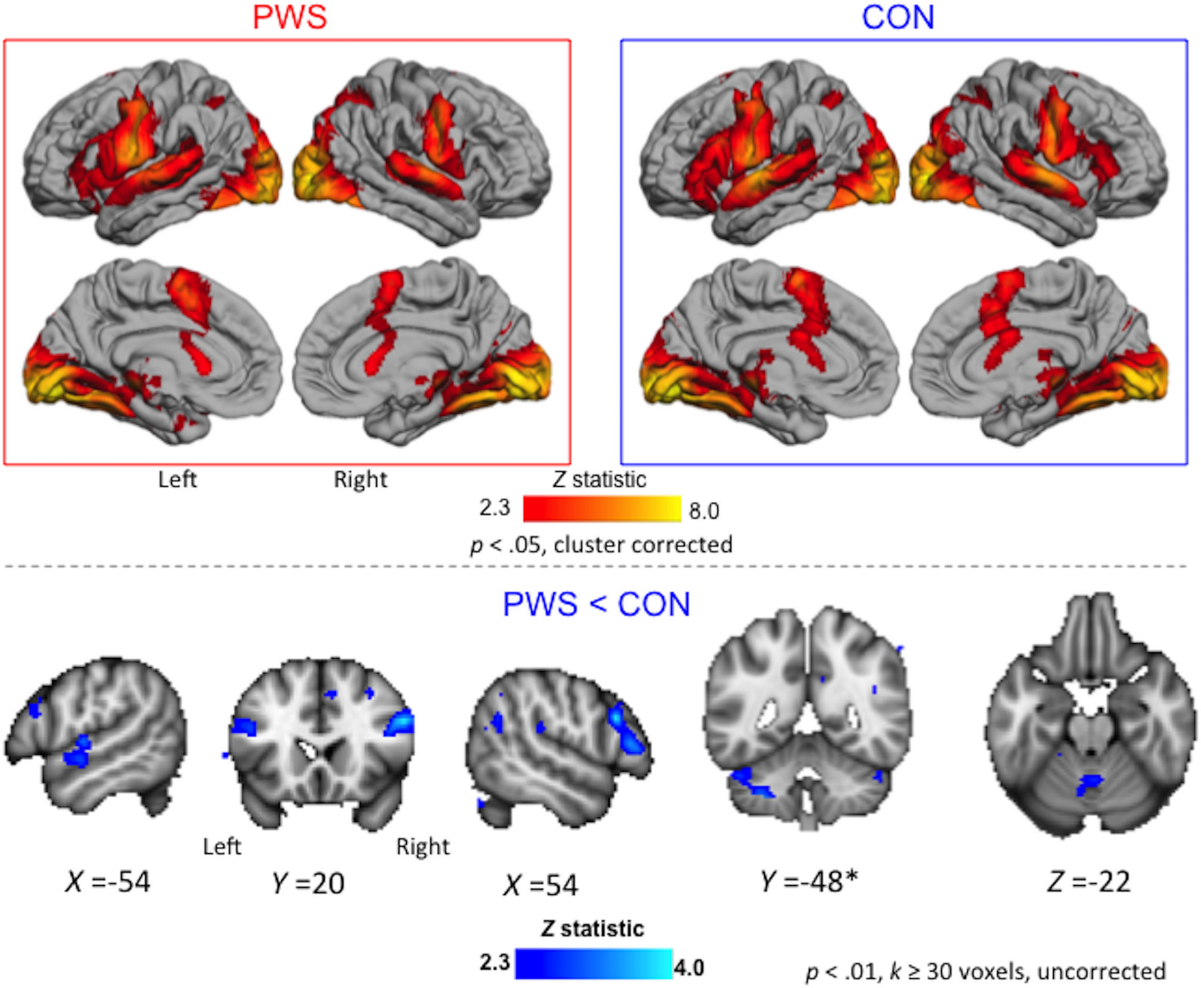
Trait effects during fluent speech. Averaged brain activity across both speech tasks is shown for the PWS and CON groups (boxed images, top; see legend to Figure 3 for details). Areas with reduced activity in PWS relative CON are shown in blue overlaid on sections through the MNI‐152 average brain (bottom; exploratory threshold of *p* < .01, *k* > 30 voxels, uncorrected; coordinates in MNI space). These differences were observed in the auditory cortex and lateral cerebellum of the left hemisphere, in inferior frontal gyrus bilaterally, and in the vermis of the cerebellum. *The left lateral cerebellum also showed an interaction effect with task. There were no areas where activity for PWS > CON at the exploratory threshold [Color figure can be viewed at http://wileyonlinelibrary.com]

During fluent speech production, there was reduced activity in PWS relative to CON at the exploratory threshold in several regions; there were no regions that showed greater activity in PWS relative to CON at this threshold (Figure 4; Table 3). Areas showing reduced activity in PWS relative to CON included the posterior parts of the IFG bilaterally, the right postcentral gyrus, the right parietal operculum, the left superior temporal cortex, the medial occipital cortex, and several portions of the cerebellum both medially and laterally. These areas were all activated above baseline in either the CON or the PWS group or both (see Table 3). There were differences in other regions but these were not activated above baseline in either group and thus represent differences in the degree to which they were deactivated in the two groups. These regions included dorsal and medial portions of the right frontal cortex, the cingulate gyrus and the angular gyrus bilaterally.

**Table 3:** Trait effects

Brain region	Number of voxels	*Z* statistic	*X*	*Y*	*Z*	PWS	CON
*Regions that were positively activated in CON or PWS or both*							
Right inferior frontal cortex	781						
Right pars triangularis		3.93	60	30	10	**−**	+
Right pars opercularis*		3.86	54	20	28	**−**	+
Right paracingulate gyrus	36	2.87	8	22	46	**0**	+
Left inferior frontal gyrus, pars opercularis*	118	2.91	−44	18	24	+	+
Right middle frontal gyrus	44	2.65	36	0	54	**0**	+
Left superior temporal cortex	283						
Left auditory cortex (Heschl's gyrus)		3.13	−54	−10	4	+	+
Left superior temporal gyrus		3.11	−56	−6	−6	+	+
Right postcentral gyrus	51	3.12	70	−12	26	**−**	+
Right inferior parietal cortex	287						
Right insular cortex		2.67	32	−22	18	**0**	+
Right parietal opercular cortex		3.1	50	−28	20	**−**	+
Left lateral cerebellum	565						
Left lobule VIIIa		3.19	−28	−48	−46	**−**	+
Left crus II		3.36	−36	−54	−44	**−**	+
Medial cerebellum	172						
Vermis V		2.83	2	−56	−24	+	+
Left medial lobule VI		2.63	−6	−66	−18	+	+
Right cerebellum, lobule VIIIb	64	2.62	10	−62	−44	**0**	+
Left medial occipital cortex (pericalcarine)	58	2.98	−20	−70	10	**0**	+
*Regions that were not active above baseline in either CON or PWS*							
Right frontal cortex	569						
Right superior frontal sulcus		2.98	20	52	28	**−**	**−**
Right medial frontal cortex		3.42	14	48	14	**−**	**−**
Medial frontal cortex	193						
Left subcallosal cingulate cortex*		2.51	−6	40	2	**−**	**−**
Right subcallosal cingulate cortex*		2.7	2	30	−8	**−**	**−**
Right mid‐cingulate gyrus	37	2.61	8	−10	38	**−**	**0**
Right angular gyrus*	276	3.44	42	−56	30	**−**	**−**
Left angular gyrus *	156	3.46	−46	−56	38	**−**	**−**
Right precuneous cortex	138	2.68	10	−56	30	**−**	**−**

Regions where activation during fluent speech was reduced in people who stutter (PWS) relative to fluent controls (CON) at the exploratory threshold of *p* < .01 uncorrected with greater than 30 voxels extent; there were no regions where activity was greater in PWS relative to CON at this threshold. Location of the highest peak in a cluster is given: voxelwise, *p* < .01, uncorrected with 30 voxel extent. For all effects, selected subpeaks within the large clusters are also described. The number of voxels in a cluster is listed along with the peak height and coordinates of the peak location in MNI‐152 standard space. *indicates clusters located symmetrically across hemispheres. The right‐most columns indicate the direction of group averages across both speaking tasks relative to the baseline for the peak voxel reported (“‐” = negative; “+” = positive, “0” = −1 < *Z* < 1).

#### 3.2.3 Interaction effects

During fluent speech production, a portion of the left lateral cerebellum showed an interaction between trait and task effects, at the exploratory threshold (Figure 4, *Y* = −48; Table 4). This area also showed increased activation for CON relative to PWS across tasks (see above). Examination of the activity levels in this area showed that while CON activated this region during both tasks, for PWS there was no activity during picture description and reduced activity relative to baseline during sentence reading (i.e., negative signal change).

**Table 4:** Interaction between task and trait during fluent speech: Regions where the effects of trait (differences between groups of people who stutter and fluent controls during fluent utterances) were different between the two tasks

Brain region	Number of voxels	*Z* statistic	*X*	*Y*	*Z*
Left lateral cerebellum	408				
Left lobule VIIb		2.65	−34	−46	−46
Left lobule VI		3.29	−32	−50	−32
Left crus II		2.99	−32	−60	−42
Left crus I		3.44	−44	−66	−38

See the legend of Table 3 for further details.

### 3.3 Neuroimaging results: State‐based subgroups

The PWS group was divided into two subgroups that were the most fluent (FLU, *n *=* *8), and the most dysfluent during scanning (DYS, *n *=* *9). This analysis allowed us to isolate activations related to proclivity to dysfluent states within a group of people who share the trait of stuttering. Both fluent and dysfluent sentences were retained for this analysis. The analysis revealed significant task differences common to both groups and significant subgroup differences common to both tasks (i.e., no significant interaction between task and subgroup; cluster forming threshold *Z *>* *2.3, family wise error corrected to *p < *.05).

#### 3.3.1 Task effects

Overall, across both tasks, both subgroups activated the expected network of areas involved in overt speech production, namely bilateral posterior superior temporal cortex, sensorimotor cortex at about the level of the face representation, SMA and preSMA, and left posterior IFG. As observed in our trait analysis, the task factor was significant, with increased activation for picture description relative to sentence reading throughout the shared network (for details see supplementary material).

#### 3.3.2 Subgroup effects

**Figure 5:**
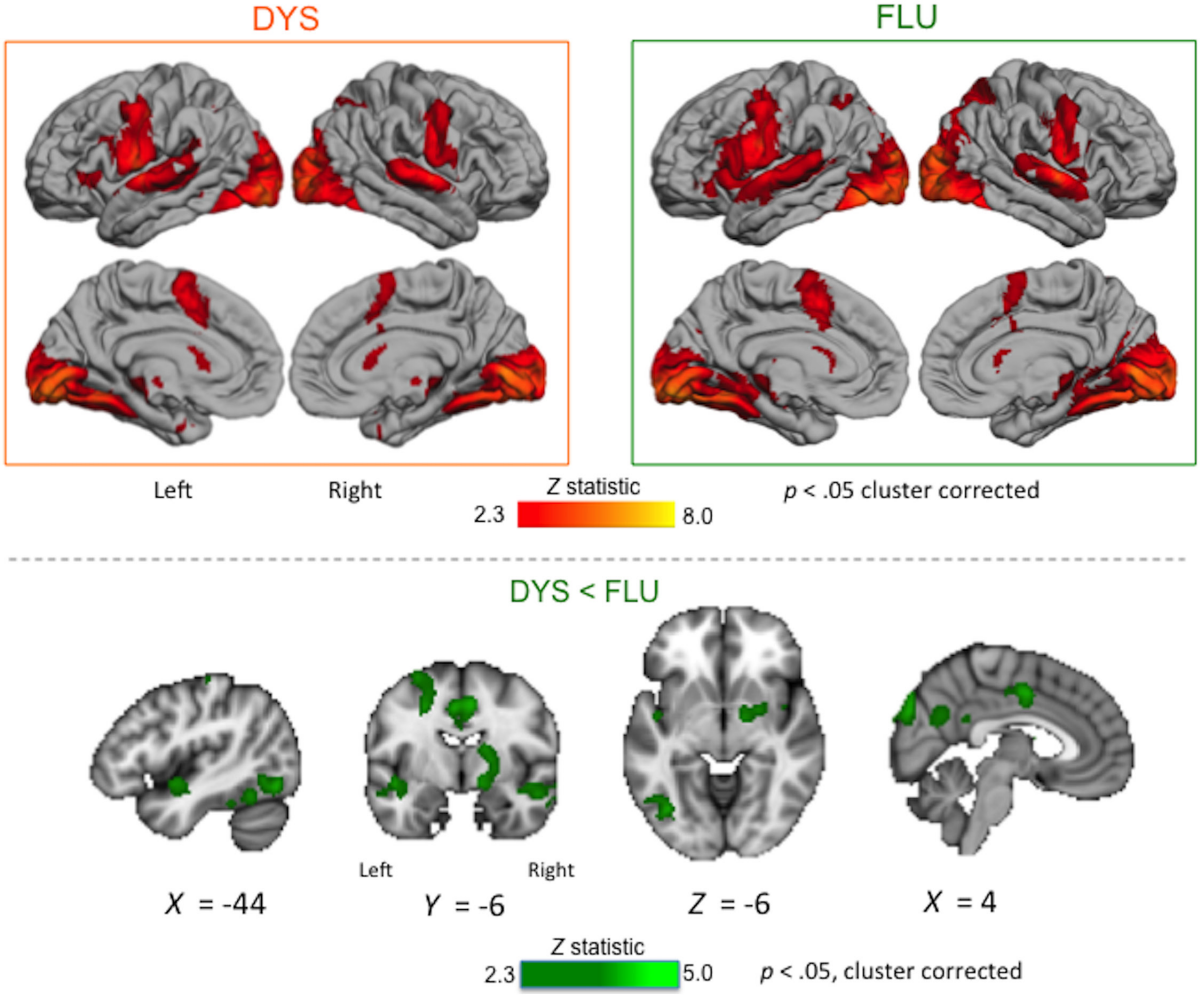
Within‐trait effects related to stuttering subgroups. Averaged brain activity across both speech tasks is shown for the DYS and FLU subgroups (boxed images, top; see legend to Figure 3 for details). Areas with reduced activity in DYS relative to FLU subgroups across tasks are shown in green overlaid on sections through the MNI‐152 average brain (bottom; *Z* > 2.3, *p* < .05, corrected; coordinates in MNI space). These differences were observed in subcortical grey matter, cingulate cortex, left inferior temporo‐occipital cortex, superior temporal cortex bilaterally, and medial parieto‐occipital cortex. There were no areas where activity was greater in the DYS relative to the FLU subgroup [Color figure can be viewed at http://wileyonlinelibrary.com]

The DYS subgroup showed reduced activity relative to the FLU subgroup in the subcortical grey matter including right caudate nucleus, putamen, and pallidum, in the cingulate cortex, the left inferior temporo‐occipital cortex, the superior temporal cortex bilaterally, and the medial parieto‐occipital cortex (Figure 5, Table 5). There were no regions showing greater activation in the DYS subgroup relative to the FLU one.

**Table 5:** Within‐trait subgroup effects during scanning in PWS

Brain region	Number of voxels	*Z* statistic	*X*	*Y*	*Z*	DYS	FLU
Right subcortical grey matter	1,413						
Right caudate nucleus		3.24	12	4	8	**0**	+
Right putamen/pallidum		3.76	20	0	−12	**−**	+
Right putamen/pallidum		2.93	22	−2	−2	+	+
Right planum polare*		2.88	44	−4	−16	**−**	**0**
Right superior temporal sulcus		3.51	52	−10	−16	**−**	+
Posterior medial‐frontal cortex	1,947						
Right mid‐cingulate gyrus*		3.67	6	−4	44	**−**	**−**
Left mid‐cingulate gyrus*		3.66	−4	−4	44	**−**	+
Right cingulate sulcus		3.91	8	−8	46	**−**	**−**
Left temporo‐occipital cortex	1,041						
Left planum polare*		3.31	−46	−6	−10	**−**	+
Left inferior temporal gyrus		3.81	−54	−40	−24	**−**	+
Left lateral cerebellum, lobule VI		2.81	−38	−50	−30	**0**	+
Left inferior temporal cortex, fusiform		3.61	−46	−54	−18	+	+
Left lateral occipital cortex		3.61	−44	−72	−12	+	+
Posterior medial cortex	1,638						
Right retrosplenial cortex		3.44	8	−46	22	**−**	**−**
Left precuneus		3.23	−2	−66	22	+	+
Right medial occipital cortex		3.84	2	−86	30	**−**	**−**
Right occipital pole		4.48	10	−96	28	+	+

Regions where there was significantly reduced activity for the DYS relative to FLU subgroups averaged across speaking tasks (*Z* > 2.3, *p* < .05 corrected). There were no regions where there was significantly more activity in the DYS relative to the FLU subgroup. The right‐most columns indicate the direction of subgroup averages across both speaking tasks relative to the baseline for the peak voxel reported (“−” = negative; “+” = positive, “0” = −1 < *Z *<* *1). See the legend to Table 3 for further details.

### 3.4 Neuroimaging results: State effects

We compared brain activity during dysfluent and fluent speech states within eight of the nine DYS individuals (one person was excluded because of a lack of fluent utterances during scanning; see Figure 2). The analysis revealed significant task differences across both fluent and dysfluent states and significant state differences across both tasks (*Z* > 2.3, *p* < .05 corrected). In addition, some state effects differed according to the task (i.e., there was a significant interaction).

#### 3.4.1 Task effects

During both fluent and dysfluent states, the expected network of areas involved in overt speech production were activated for both tasks (as above). As for the previous analyses between subjects, there was significantly more activation during the picture description task relative to sentence reading (for details see supplementary materials).

#### 3.4.2 State effects

**Figure 6:**
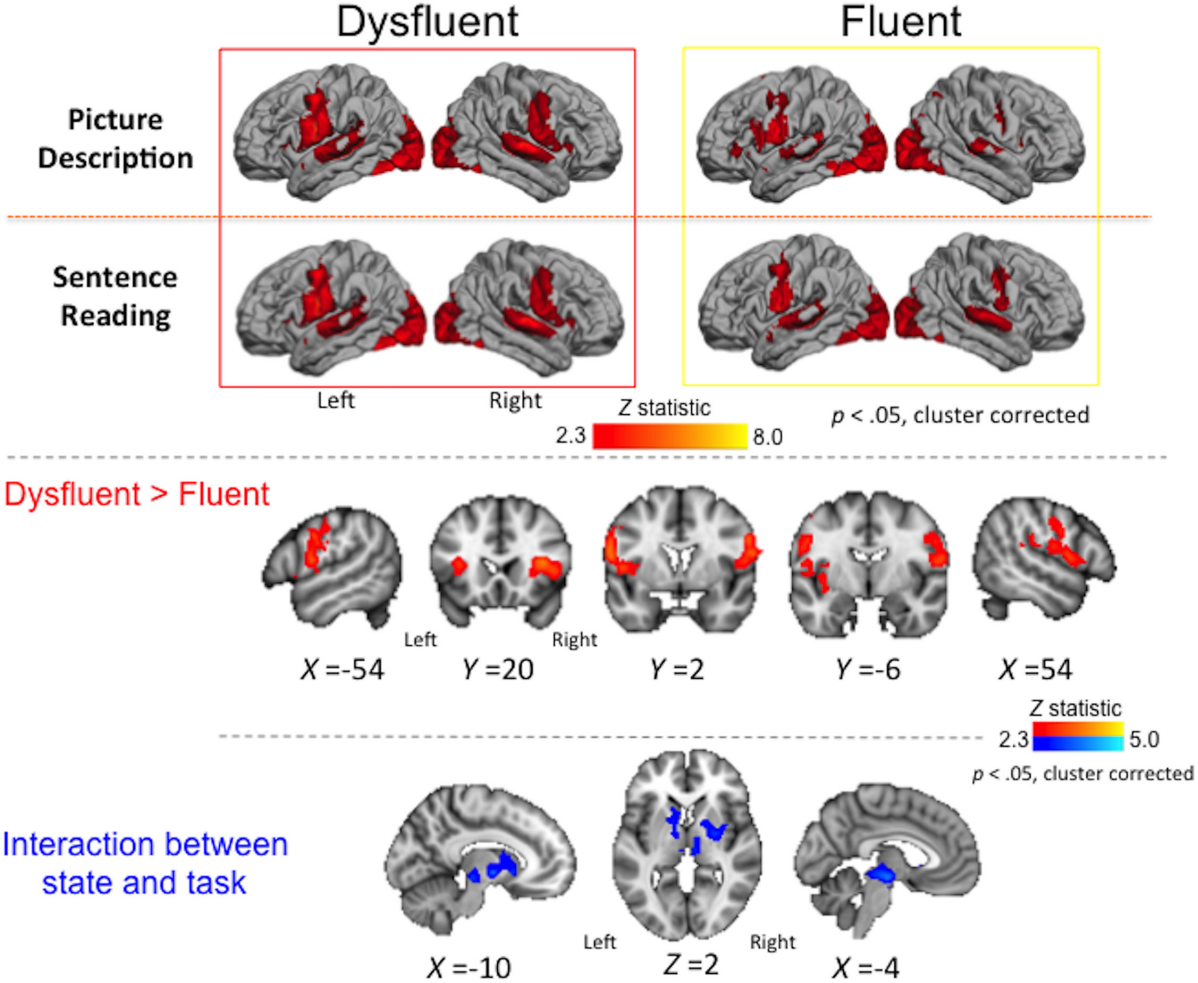
State effects. Averaged brain activity during each speech state (dysfluent and fluent) is shown for the picture description and sentence reading tasks separately (boxed images, top, see legend to Figure 3 for details). Areas showing greater activity during dysfluent relative to fluent states within individuals across tasks are shown in red (middle; see legend to Figure 5 for details). Individuals had greater activity during dysfluent relative to fluent speech in the lateral inferior frontal and premotor cortex extending into the opercular cortex and anterior insula bilaterally. Subcortical areas showing a significant interaction between state and task are shown in blue (bottom; see legend to Figure 5 for details) [Color figure can be viewed at http://wileyonlinelibrary.com]

As expected, both fluent and dysfluent speech states recruited the same network of regions averaged across tasks (Figure 6). There was significantly more activity during the dysfluent state relative to the fluent state in the IFG and premotor cortex extending into the operculum and anterior insula almost symmetrically in both hemispheres (Figure 6, Table 6). Across all these regions, both dysfluent and fluent states showed increased activation relative to baseline but activity during the dysfluent state was greater. There were no areas for which dysfluent states showed reduced activity relative to fluent states.

**Table 6:** State effects in the DYS subgroup

Brain region	Number of voxels	*Z* statistic	*X*	*Y*	*Z*
Right premotor and prefrontal cortex	2,148				
Right frontal operculum*		3.82	36	18	12
Right central operculum*		3.42	44	18	6
Right inferior frontal gyrus,pars opercularis		3.67	48	8	8
Right precentral gyrus*		3.54	58	0	28
Left premotor and prefrontal cortex	1,633				
Left frontal operculum*		3.38	−36	16	14
Left anterior insula		3.57	−30	14	8
Left central operculum*		3.54	−46	6	4
Left precentral gyrus*		3.73	−58	0	20

Regions where there were significant differences between fluent and dysfluent utterances within individuals across both tasks. See the legend to Table 3 for further details.

#### 3.4.3 Interaction effects

As noted above, the activation during the two states differed significantly according to task. The areas showing this significant interaction were located subcortically in dorsal striatum bilaterally (left caudate nucleus and right putamen), the midbrain, at the level of the substantia nigra, extended anteriorly into posterior thalamus and external pallidum, bilaterally (Figure 6, Table 7). Examination of the mean activity in these areas indicated reduced activity during fluent picture description and dysfluent sentence reading relative to the baseline and the opposite state (*i.e*. negative signal change across these areas).

**Table 7:** Interaction between task and state in DYS subgroup: Regions where the effects of state (differences between fluent and dysfluent utterances) were different between the two tasks within individuals

Brain region	Number of voxels	*Z* statistic	*X*	*Y*	*Z*
Subcortical grey matter	1,738				
Left nucleus accumbens		2.85	−10	14	−4
Left caudate nucleus		2.77	−12	10	6
Left pallidum*		3.26	−10	−4	−4
Right putamen		2.84	26	−6	4
Right pallidum*		2.74	14	−8	−2
Left dorsal midbrain		3.28	−4	−16	−12
Left thalamus*		2.58	−6	−22	−2
Right thalamus*		2.83	8	−22	0

See the legend to Table 3 for further details.

## 4 DISCUSSION

In the current study, we used a hierarchical approach to directly isolate fMRI activity related not only to general traits of stuttering but also to dysfluent states of speech within people who stutter. We also distinguished specific state effects within individuals who stutter from activation related to the frequency of dysfluent speech produced in the scanner by different subgroups of people who stutter. Furthermore, we examined whether any of these factors differed according to the task performed in the scanner. The results indicated differences in brain activity in stuttering that can be related to four factors: general traits, frequency of dysfluent states, specific speech states, and task demands. Below we elaborate on each of these factors before discussing the task findings from the study and some methodological considerations.

### 4.1 General trait effects of stuttering

Our trait analysis failed to reveal differences in brain activity across tasks in people who stutter relative to fluent controls at a statistical threshold corrected for multiple comparisons. We reported results instead at an exploratory threshold that did not require such a large extent of voxels to be activated to pass the corrected significance threshold. At this more lenient threshold, people who stutter showed reduced activity relative to controls in the posterior parts of the inferior frontal gyrus bilaterally, the right postcentral gyrus, the right parietal operculum, the left superior temporal cortex, the medial occipital cortex, and several portions of the cerebellum both medially and laterally. These trait effects were predominantly due to greater activity during task relative to baseline in the control group compared with the stuttering group (see Table 3). Several of these regions also showed reduced activity in people who stutter in previous reports: inferior frontal cortex (Neef et al., 2016; Watkins et al., 2008); left superior temporal (auditory) cortex (e.g., Brown et al., 2005; Giraud et al., 2008; Watkins et al., 2008;Toyomura et al., 2015;Budde et al., 2014); and medial cerebellum (Chang, Kenney, Loucks, & Ludlow, 2009; Ingham et al., 2012). However, our findings are notably inconsistent with the broader claim that *overactivity* of the right inferior frontal cortex and cerebellar vermis are “neural signatures” of stuttering (Brown et al., 2005; Belyk et al., 2017; Budde et al., 2014).

It is possible that we did not see areas of overactivity in people who stutter in our study because the analysis was restricted to only fluent utterances. The overactivity of the right inferior frontal cortex, in particular, has recently been described to reflect overactive inhibition of speech responses (Neef et al,. 2016) that would occur most often during the dysfluent state. Consistent with this interpretation is the overactivity of the right inferior frontal cortex seen in the state analysis for the dysfluent relative to the fluent state (see Figure 6).

The left inferior frontal cortex was underactive in our study, consistent with findings from one meta‐analysis identifying underactivity in left ventral premotor cortex as a stuttering trait (Belyk et al., 2017). According to an influential model of speech production, the left inferior frontal cortex is thought to be important for feedback monitoring and the release of speech plans for feedforward models (Guenther & Ghosh, 2003). Relatedly, the specific location of trait effects in the cerebellar vermis reported here was close to somato‐motor representations for the articulators that are theorized to contain copies of cortical maps (Buckner, Krienen, Castellanos, Diaz, & Yeo, 2011). These maps too are thought to be utilized in both feedback monitoring and feedforward models for speech production (Guenther & Ghosh, 2003). Another node in this speech network was also found to be underactive in people who stutter, namely the left primary auditory cortex. The auditory cortex receives external feedback and is thought to contain target and error maps used to update internal speech plans (Guenther & Ghosh, 2003; Tourville & Guenther, 2011). To summarize, our analysis of fluent speech in people who stutter has revealed a pattern of reduced activity relative to controls in a network of brain regions thought to be critical for feedback‐based speech monitoring. One tentative interpretation of this pattern is that it reflects a compensatory gating mechanism reducing signal throughout the feedback control system, which in turn could counter an over‐reliance on feedback that is theorized to cause stuttering (Max, Guenther, Gracco, Ghosh, & Wallace, 2004).

We also observed trait differences in the left lateral cerebellum in subregions functionally connected to prefrontal cortex (e.g., left crus I) (O'Reilly, Beckmann, Tomassini, Ramnani, & Johansen‐Berg, 2010). People who stutter showed negative signal change in the lateral cerebellum, while fluent controls showed positive activation relative to baseline. Furthermore, trait‐related activation in this region interacted with task: fluent controls recruited this region to a greater extent for picture description than for sentence reading, whereas people who stutter did not. Similar effects were observed in this region during PET imaging of longer speech utterances in people who stutter (Ingham et al., 2012) and altered functional connectivity between the lateral cerebellum and premotor cortex was also reported in stuttering (Lu et al., 2010). Abnormal activity in the lateral cerebellum could reflect maladaptive prediction processing related to speech and language planning (Sokolov, Miall, & Ivry, 2017). We speculate that during fluent speech in people who stutter, abnormal activity in this region reflects a successful compensatory mechanism involving disengagement. The degree to which regions must disengage may be moderated by task demands, which would explain the interaction between task and trait in this study. A “disengagement” that resulted in compensation would also be consistent with reports of increased baseline activation of the cerebellum in people who stutter (Ingham et al., 2012). Future work is needed to investigate the role of cerebellar activation at rest as it relates specifically to trait and state effects in stuttering in order to better clarify the nature of effects observed in our study.

### 4.2 Effects related to the frequency of dysfluent utterances

The findings from our study replicated several previously reported associations between the frequency of the dysfluent speech and activity in the basal ganglia (Braun et al., 1997; Giraud et al., 2008; Ingham et al., 2004, 2012; Kell et al., 2009; Toyomura, Fujii, & Kuriki, 2011), cingulate cortex (Budde et al., 2014; Ingham et al., 2012), inferior temporo‐occipital cortex (Fox et al., 2000; Giraud et al., 2008; Ingham et al., 2004), and superior temporal cortex (planum polare), occurring bilaterally (Kell et al., 2009). All of these regions showed reduced activation in participants who showed higher frequency of dysfluency during our study.

In the contrast of subgroups of people who stutter, we observed significant increases in striatal activity for the subgroup of more fluent individuals. Previous research has concluded that increases in activity in this part of the basal ganglia reflect treatment success (Ingham, Wang, Ingham, Bothe, & Grafton, 2013). This categorization is based on the observation that activity typically increases from pre‐ to post‐intervention (Giraud et al., 2008; Neumann et al., 2003; Toyomura et al., 2015), and also during fluency enhancement tasks (Toyomura et al., 2011). Based on these observations, it is possible that our stuttering subgroups differed because of some undetected long‐term effect of treatment, or that one group successfully used the fluency enhancing properties of the scanner (masking noise and rhythm) more than the other. The possibility remains, however, that these are spontaneously occurring different subgroups within the stuttering population. Identification of subgroups within the stuttering population that differ not just in frequency but also type of speech dysfluency is an important next step.

An alternative explanation of the observed greater activity in the striatum in the fluent subgroup is that the activation is the result of the fluent speech, not the cause. The logic can be simply stated as follows: fluent speech is more rewarding than dysfluent speech for people who stutter. The “expectation of success” for movements results in rewards (e.g., boost of dopamine), and this function is processed in the striatum (O'Reilly, Jbabdi, Rushworth, & Behrens, 2013). The models necessary for planning dynamic action, such as the coordination required for speech execution, utilize the putamen (O'Reilly et al., 2013). Expectation of reward would be specific to fluent speech activation and increase levels relative to activity for dysfluent speech.

One of most common findings during both PET and fMRI imaging of sequential speech in stuttering is overactivity of medial premotor cortex (preSMA/SMA; Braun et al., 1997; Fox et al., 1996, 2000, Ingham et al., 2000, 2012; Kell et al., 2009; Preibisch et al., 2003; Sakai et al., 2009). Activation of medial premotor cortex is also related to stuttering frequency and is theorized to reflect task effects in stuttering (Ingham et al., 2012). The only nearby region showing activation differences in our study was found inferior to the SMA complex, in the cingulate gyrus, occurring bilaterally, which showed greater activity in the mostly fluent subgroup. These differences appeared to be due to large, negative signal change in the dysfluent subgroup, whereas the fluent subgroup showed positive signal change relative to baseline in the left cingulate gyrus. The basal ganglia are functionally connected to the medial frontal cortex in a circuit thought to be involved in the preparation and execution of movements (Cunnington, Windischberger, Deecke, & Moser, 2002). The subgroup effect observed could reflect inefficient coordination of that network via connections through the cingulate cortex.

Our study also adds to reports of significant relationships between temporo‐occipital cortex activation and the frequency of dysfluency in stuttering (Brown et al., 2005; Fox et al., 2000; Giraud et al., 2008; Ingham et al., 2004). We observed greater activity in visual association areas in the more fluent subgroup in the left inferior temporo‐occipital cortex and medial parieto‐occipital cortex. The relationship between left hemisphere fusiform cortex activity and stuttering severity has been observed during resting state fMRI (Sitek et al., 2016) as well as in speech tasks as part of a treatment study, in which the effect did not attenuate with therapy (Giraud et al., 2008).

The greater activity of these ventral areas in the fluent subgroup was located in regions specialized for processing word and object recognition and show interactive effects with other language processes (e.g., phonology and semantics; see Price 2012). Differences in activity levels in these regions during our tasks, which required picture description and reading, could reflect greater influences in the fluent subgroup of visual imagery or top–down processes from higher‐order language areas. Why this effect would be more obvious in the more fluent subgroup and not in the dysfluent one requires further investigation.

Finally, we replicated previous associations of significant relationships between activity in planum polare and the frequency of dysfluent speech in stuttering (Kell et al., 2009). In our study, in both hemispheres the average signal change for the more dysfluent subgroup was negative relative to baseline, and larger than the positive changes relative to baseline observed in the fluent subgroup. The interpretation of such “deactivations” relative to “activations” is not straightforward. However, our findings could reflect a successful recruitment of the planum polare for integration of feedback into speech motor programs, which is somewhat compensatory for altered premotor activity observed during dysfluent states directly (see below).

### 4.3 Specific state effects within stuttering individuals

Identification of state effects, specifically those associated with dysfluency in people who stutter is of major importance. There is surprisingly little data on this state (see Section 1), with many reports relying instead on correlations with stuttering severity that might be better described as “proclivity to stutter” and more akin to our frequency analysis described above. The reason for the paucity of data on the dysfluent state is due to the fact that it occurs relatively infrequently in most individuals, particularly when in the scanning environment, which also can be fluency enhancing. Obtaining a maximum of 160 utterances during scanning in our study allowed us to compare a sufficient number of fluent and dysfluent utterances (at least 10 of each in each run) in a subgroup of 8 individuals. Within these individuals, we directly compared activation during dysfluent speech states to that underlying fluent speech states.

Activity was greater in the lateral inferior frontal and premotor cortex, frontal operculum and anterior insula bilaterally during dysfluent relative to fluent speech. Importantly, we further confirmed that these effects were not related to head motion in the scanner, which did not differ between these speech states. Our findings are largely consistent with previous reports of overactivity in inferior frontal and premotor cortex in stuttering (Braun et al., 1997; Ingham et al., 2012), including in the central opercular cortex (Fox et al., 1996, 2000; Giraud et al., 2008; Kell et al., 2009; Neumann et al., 2003, 2005; Preibisch et al., 2003; Sakai et al., 2009; Toyomura et al., 2015). In particular, we observed overactivity in the right inferior frontal cortex extending into the opercular cortex and anterior insula, which has been previously described as a “neural signature” of people who stutter (Belyk et al., 2017; Brown et al., 2005; Budde et al., 2014). It is worth noting, however, that the majority of the previous reports did not isolate the dysfluent state and report this overactivity as a trait effect (difference between people who stutter and controls).

Generally, the increased recruitment of right inferior frontal cortex was interpreted as compensatory in stuttering based on higher post‐treatment activation in the region in people who stutter (MNI coordinates: 48,14,4; Neumann et al., 2003, 2005; Preibisch et al., 2003). Furthermore, the compensatory nature of the right hemisphere overactivity was often theorised to reflect a response to a structural deficit in the left hemisphere, as previously described in diffusion imaging studies of people who stutter. However, we have found that these structural differences are often bilaterally distributed (Connally et al., 2014; Watkins et al., 2008), as were the state effects in the current study. Even though we observed increased activation in the same right posterior inferior frontal region very near those previously reported to be sensitive to therapy (MNI coordinates: 44, 18, 6), the increased activation was also seen on the left during dysfluency. Furthermore, we did not observe these increases in the group of people who stutter when compared with fluent controls during fluent speech. This makes it difficult for us to conclude that the activity in this right hemisphere region reflects a compensatory process.

As noted above in the discussion of general traits, none of these regions overactive during the dysfluent state were overactive during the fluent speech of people who stutter relative to the controls, and no regions showed overactivity during fluent states relative to dysfluent states within individuals. The idea that the right hemisphere overactivity seen here during the dysfluent state and reported previously in other group analyses relates to an inhibitory or stopping process seems to be the best explanation of this consistently reported result (see Neef et al., 2016). This “stopping” response is right lateralised even for inhibition of speech (Xue, Aron, & Poldrack, 2008). The left hemisphere overactivity of the same regions in the dysfluent state requires a different explanation, therefore. This could be compensatory or reflect the greater speech effort that comes with the dysfluent state. Whatever the explanation, the symmetrical pattern of overactivity during the dysfluent state of brain areas involved in speech motor planning and execution and their right hemisphere homologues is striking.

One notable unilateral state effect was observed in the right parietal operculum. This was also the only region that indicated both state and trait effects in our study. Activation was reduced in this region for fluent speech states relative to dysfluent speech states, and in fluent speech in people who stutter relative to fluent controls, across tasks. Further, the stuttering group showed reduced activation of this region relative to baseline during fluent speech, while the fluent controls showed a positive signal change. The parietal operculum is thought to house the secondary somatosensory cortex, with representations for the face in addition to other body maps (Eickhoff, Schleicher, Zilles, & Amunts, 2006). One might speculate that increased activation of this region during the dysfluent state reflects inefficient sensory feedback processing (Tourville, Reilly, & Guenther, 2008). Such a scenario would be consistent with a general need to reduce feedback‐based processing in order to be fluent, which fits the general pattern observed in our trait analysis.

The state analysis revealed an interaction with task. That is, subcortical areas activated differently during dysfluency relative to fluency depending on the task being performed. We previously discussed the task‐dependent trait effect in the cerebellum. Here, task‐dependent state effects were observed extending from the dorsal striatum to the midbrain and were due to negative activity (relative to baseline) for fluent states during picture description and dysfluent states during sentence reading. Such relative differences in effectively “deactivations” are difficult to interpret. Nevertheless, they point to a potentially complex role for the basal ganglia in stuttering that is task dependent. Further investigation could aid interpretation of previously inconsistent findings of basal ganglia activity in stuttering research. For example, previous reports indicated increased basal ganglia activity was associated with increased stuttering severity (Giraud et al., 2008; Ingham et al., 2012), whereas increased activity in the same regions was associated with enhancement of fluency in other reports (Toyomura et al., 2011), and the putamen and midbrain in particular were hyperactive during fluent sentence reading across several tasks (Watkins et al., 2008). It is notable also that none of the meta‐analyses has described basal ganglia abnormalities.

According to theory (Alm, 2004), one potential outcome of abnormal basal ganglia function, or a dopaminergic imbalance therein, is a delay in motor signals in the putamen‐SMA motor execution loop that in computational models results in simulated stuttering (Civier, Bullock, Max, & Guenther, 2013). In our participants, the disengagement needed for fluent speech (in the case of picture description, for example) could reflect efforts to force movement into the next portion of a speech sequence involving disrupted loops with inferior frontal regions (Guenther & Ghosh, 2003). We observed the predicted net increase in activity in inferior frontal regions during dysfluent states, and a net decrease during fluent speech in stutterers relative to fluent controls in nearby cortex, which would be consistent with a “disengage for fluency” scenario. On the other hand, during sentence reading, the decreased subcortical activity for dysfluent speech could reflect the theorised delay in the putamen‐SMA loop itself which results in insufficient “go” signals to initiate movements (Civier et al., 2013). Because the putamen can play both inhibitory and excitatory roles through connections (via the pallidum and thalamus) with the SMA and substantia nigra for the planning of volitional movements, distinguishing between these possibilities with imaging studies is not possible. Notably, a portion of the right hemisphere pallidum showing the state by task interaction was also associated with differences in the frequency of dysfluent utterances between stuttering subgroups.

### 4.4 The separation of trait, state, and frequency effects

**Figure 7:**
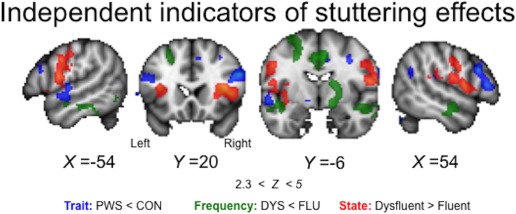
Summary of general trait, specific state, and frequency effects. Areas with reduced activity in PWS relative to CON during fluent speech are shown in blue (trait effects). Areas with reduced activity in the DYS relative to the FLU subgroup of stutterers are shown in green (frequency effects). Areas with increased activity during dysfluency relative to fluency within individuals in the DYS subgroup are shown in red (state effects). The regions showing these effects are mostly spatially distinct. Coloured statistical maps are overlaid on sections through the MNI‐152 average brain (*Z* > 2.3, *p* < .05, corrected, coordinates in MNI space) [Color figure can be viewed at http://wileyonlinelibrary.com]

In the current study, we demonstrated that abnormal activation in regions reflecting general traits of stuttering could be separated from those reflecting differences between dysfluent and fluent speech states (Figure 7). Activity related to the differences between speech states was also found in regions separate to those showing activity related to stuttering frequency (i.e., that differed between subgroups of people who stutter). The general dissociation of state, trait, and frequency effects in stuttering was consistent across tasks. Interestingly, when speaking fluently, activity in people who stutter was generally reduced relative to that in controls. But, people who stutter who are more fluent during scanning, showed a general pattern of overactivation relative to those who were dysfluent. In this case, the overactivation could be considered causal to achieving fluency in the scanner in this relatively fluent subgroup or, conversely, failure to activate these regions in the dysfluent subgroup is the cause of their more frequent dysfluency. In the state analysis, a different pattern emerged in a separate set of brain regions. That is, brain areas were generally overactive in the dysfluent state compared with the fluent state within people who stutter. Here, the interpretation might be different to that above, and different for different brain regions. The overactivity could be considered to cause the speech dysfluency, be the consequence of it (either as a correlate of greater inhibition or error responses) or reflect an attempt to overcome it. It would be possible to test some of these hypotheses using interference techniques such as brain stimulation.

Overall, the distinctions in the brain areas involved in state and trait analyses described here are somewhat consistent with the conclusions of two meta‐analyses attempting the same dissociation (Belyk et al., 2017; Budde et al., 2014). There are notable differences also. For example, only one brain region showed abnormal activation that indicated both trait and state effects, namely the right parietal operculum, which was underactive in people who stutter in the trait analysis and overactive during dysfluency in the state analyses. Subcortically, activity in the right putamen/pallidum was associated with frequency of dysfluent speech states, and also with the interaction between state and task. Furthermore, we observed that abnormal activation of subcortical structures in stuttering differed according to task. These interactions with task were observed: (1) in the left lateral cerebellum reflecting trait effects and (2) in the basal ganglia, thalamus, and midbrain reflecting state effects. The spatial separation of these different effects may aid interpretation of the results of other analyses in which it has not been possible to separate them.

### 4.5 Task effects

In this study, we used two tasks: picture description and sentence reading. The amount of dysfluency occurring during these different tasks was highly variable among stuttering individuals but did not consistently differ on average across the group. The picture description task involves generating speech content (i.e., cognitively and linguistically selecting the semantic content, morphology, and syntactic frame for a sentence). In contrast, sentence reading does not involve these processes and instead requires the reader to produce prescribed speech content, which means that words cannot be avoided or substituted. Such strategies are commonly employed by people who stutter to avoid “difficult” words on which they predict dysfluency. Both tasks require retrieval and production of the phonology and articulatory processes for speaking either the provided or the self‐generated sentence that are arguably matched.

The imaging data for these tasks shows very similar networks of activity in lateral and medial cortical areas involving left inferior frontal cortex, the ventral premotor and sensorimotor cortex, the SMA complex on the medial surface, the superior temporal cortex and occipital cortex, bilaterally (see Figure 3). The additional cognitive and linguistic effort required by the picture description task relative to the sentence reading one led to the expected greater activation of the brain network of areas involved in these tasks. In particular, as evident in Figure 3, picture description activates the left inferior frontal cortex to a greater extent than does sentence reading, presumably reflecting the additional requirements for lexical search and retrieval and generation of the syntactic frame in the former. Similar selection processes presumably drive the greater activity on the medial surface in the SMA for picture description. The contrast of these tasks was not the main focus of the study but the effects described are consistent with findings from the large literature using similar tasks (see review by Price, 2012; Brown et al., 2005). The details of these differences for each of the analyses is provided in supplementary material. The brain areas showing an interaction of tasks with trait or state effects have been discussed previously (see above).

### 4.6 Methodological considerations

In this study, we implemented some data acquisition approaches aimed at clarifying state and trait effects in stuttering imaging research. Overt speech production during the two tasks was recorded for off‐line analysis. This allowed us to assess compliance of all participants with task performance as well as evaluating dysfluency during scanning. To our surprise, some participants (controls and people who stutter) were not compliant with task instructions and introduced unexpected behavior. In some cases, whole datasets were eliminated from analyses. In other, less severe cases, stimuli could be reallocated to the correct task or individual epochs removed from analysis. This approach ensured greater accuracy in our analyses.

In addition, the in‐scanner recordings allowed us to determine which utterances contained speech dysfluency and which were fluent. Trial‐by‐trial precision enabled an accurate trait analysis of fluent speech in the whole group and a state analysis in the subgroup of people who were dysfluent during scanning. Nevertheless, this analysis was limited in that we only examined activation following a speech event, and it could be the case that an error signal detected before trials gives us a causal indicator of dysfluent states. Use of real‐time fMRI during the dysfluent state may be informative in terms of determining whether brain activity is compensatory, reflecting error detection, or part of an inhibitory response. Furthermore, we did not distinguish between typical or atypical dysfluencies within our state analysis (Jiang et al., 2012), nor did we look at activity related only to a specific sort of typical dysfluency (e.g., blocks, Sowman et al., 2012). This is in part due to difficulty in knowing whether a normal speech dysfluency, such as repetition of whole phrases or multi‐syllabic words or interjections such as “um” and “er” are part of stuttering behavior. These dysfluencies occur commonly in the speech of people who stutter but do not “count” in standard measures of stuttering frequency (e.g., SSI). The other reason we could not distinguish among types of dysfluency in this study was simply one of practicality, as there were insufficient numbers of epochs containing only stuttering dysfluencies without normal dysfluencies such as interjections, and vice versa. Again a real‐time fMRI or continuous imaging approach could aid such analyses. Finally, although we used a binary approach to this analysis it is likely that a parametric approach that looked at the amount or type of dysfluency could prove useful in future studies.

As noted previously, it is quite likely that the dysfluent state is more commonly accompanied by head movements that could in turn introduce noise into the brain images. Our study dealt with this potential confound by using a sparse‐sampling design in which only the peak of the haemodynamic response is measured during the image acquisition. This peak occurs some 4–6 seconds after an event. The timing of our image acquisition was intended to capture responses to the speech event that had occurred in the previous silent period between acquisitions. It is unlikely that the head would be moving during the image acquisition, therefore, and participants were explicitly instructed to stop speaking when the fixation cross appeared. Even so, to evaluate the potential for head movements to occur more frequently during the dysfluent state, we compared the degree to which each image acquisition needed to be corrected for motion in those images that followed fluent and dysfluent utterances and the number of motion outliers that were detected in each state. There were no significant differences between the two states in either of these measures, allowing us to conclude that motion artefacts related to speech dysfluency have not contaminated our data.

Finally, by implementing two different tasks, we were able to explore whether the patterns of abnormal brain activity seen in trait and state analyses vary depending on the task. We found limited, but some evidence for this effect. It would be interesting to explore this further with a greater range of speaking tasks, including natural and induced speech fluency, as well as spontaneous and imitated speech dysfluencies.

## CONCLUSION

We conclude that indicators of traits, states, and relative frequency of dysfluency in stuttering are potentially spatially‐separable factors. Furthermore, we showed that task demands can influence these effects. In particular, task demands might explain the complex role played by subcortical control structures in stuttering and speech production more broadly. Overall, our findings support efforts to distinguish state and trait effects directly within a study sample, and provide evidence of subgroups of people who stutter who differ in frequency of dysfluent speech in the scanner. The results point to the need for the use of different tasks that elicit longer speech utterances so that future work can expand on the neural correlates of different dysfluent states.

## CONFLICTS

MJ receives royalty payments from the commercial licensing of FSL software (though academic and non‐commercial use, such as that for this manuscript, is free). No other authors have conflicts to report.

## Supporting information

Additional Supporting Information may be found online in the supporting information tab for this article.

Supporting Information Figure 1

Supporting Information Figure Legend

Supporting Information Table I

Supporting Information Table II

Supporting Information Table III

Supporting Information
