# Supplementary figures and images for: Separation of trait and state in stuttering
Source: Hum Brain Mapp. 2018 Apr 6;39(8):3109–26. doi: 10.1002/hbm.24063 (PMC6055715; doi:10.1002/hbm.24063)

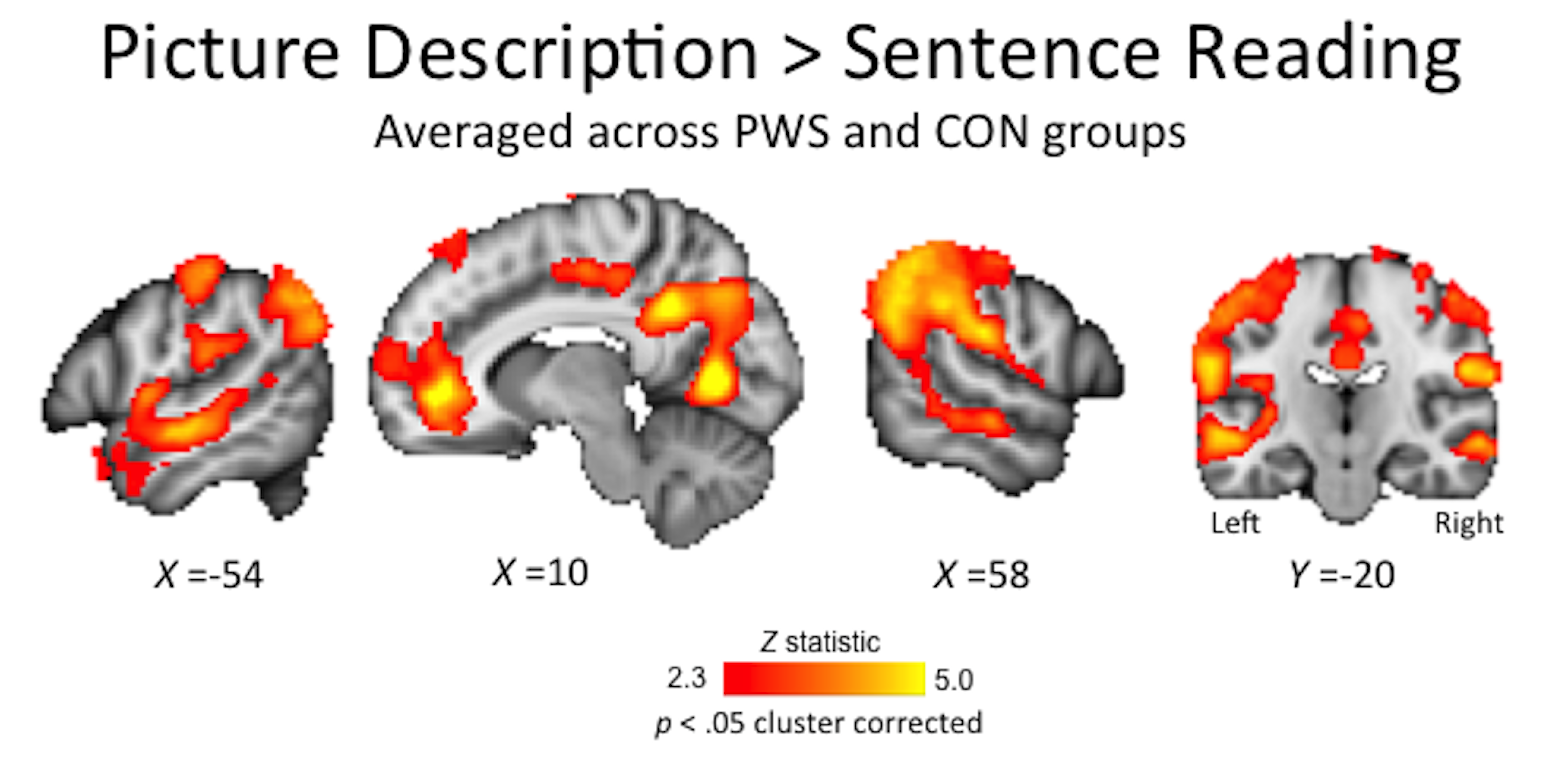

Supplement: Supplementary file 1 — Supporting Information Figure 1 [file HBM-39-3109-s001.tiff]
