# Supplementary material for: Separation of trait and state in stuttering
Source: Hum Brain Mapp. 2018 Apr 6;39(8):3109–26. doi: 10.1002/hbm.24063 (PMC6055715; doi:10.1002/hbm.24063)
Supplement: Supplementary file 2 — Supporting Information Figure Legend [file HBM-39-3109-s002.docx]

Supplementary Figure 1. Task effects during fluent speech. Areas with greater activity during picture description relative to sentence reading are shown in red-yellow overlaid on sections through the MNI-152 average brain (Z > 2.3, P < .05, corrected; coordinates in MNI space). The direct contrast of tasks across both PWS and CON groups resulted in increased activation throughout the speech-motor network for picture description relative to sentence reading.
