# Supplementary material for: Separation of trait and state in stuttering
Source: Hum Brain Mapp. 2018 Apr 6;39(8):3109–26. doi: 10.1002/hbm.24063 (PMC6055715; doi:10.1002/hbm.24063)
Supplement: Supplementary file 3 — Supporting Information Table I [file HBM-39-3109-s003.docx]

Supplementary Table I: General task effects. Regions where there significantly greater activity during picture description relative to sentence reading.

| Brain region | # voxels | Z statistic | X | Y | Z |
| --- | --- | --- | --- | --- | --- |
| Right medial and orbitofrontal cortex | 4051 |  |  |  |  |
| Right inferior frontal (orbitalis) |  | 3.99 | 40 | 48 | 4 |
| Right paracingulate gyrus |  | 5.53 | 10 | 44 | -2 |
| Right cingulate gyrus (anterior)* |  | 4.52 | 2 | 40 | 8 |
| Left cingulate gyrus (anterior)* |  | 5.3 | -8 | 38 | 4 |
| Right orbitofrontal gyrus |  | 4.15 | 20 | 38 | -10 |
|  |  |  |  |  |  |
| Right lateral frontal cortex | 1275 |  |  |  |  |
| Right middle frontal gyrus |  | 3.75 | 52 | 24 | 44 |
| Right superior frontal gryus |  | 3.46 | 14 | 34 | 58 |
|  |  |  |  |  |  |
| Left lateral peri-Sylvian cortex | 10134 |  |  |  |  |
| Left central operculum |  | 4.76 | -40 | -8 | 2 |
| Left dorsal precentral gyrus |  | 4.81 | -34 | -14 | 68 |
| Left superior temporal sulcus |  | 4.69 | -52 | -14 | -10 |
| Left supramarginal gyrus* |  | 5.27 | -60 | -24 | 22 |
| Left dorsal postcentral gyrus |  | 4.69 | -42 | -32 | 64 |
| Left angular gyrus* |  | 4.77 | -52 | -66 | 46 |
|  |  |  |  |  |  |
| Right inferior parietal cortex | 9782 |  |  |  |  |
| Right supramarginal gyrus* |  | 4.98 | 58 | -22 | 26 |
| Right angular gyrus* |  | 5.17 | 60 | -50 | 52 |
|  |  |  |  |  |  |
| Posterior medial cortex | 6178 |  |  |  |  |
| Right retrosplenial cortex* |  | 5.28 | 8 | -48 | 32 |
| Left retrosplenial cortex* |  | 4.45 | -6 | -50 | 32 |
| Right medial parietal cortex |  | 4.38 | 8 | -66 | 38 |
| Right pericalcarine cortex |  | 6.12 | 6 | -68 | 4 |

Location of the highest peak in a cluster is reported, Z > 2.3 cluster forming threshold, FWE corrected to P < .05 Selected subpeaks within the large clusters are also described. The number of voxels in a cluster is listed along with the peak height and coordinates of the peak location in MNI–152 standard space. ^*^indicates clusters located symmetrically across hemispheres.
