# Supplementary material for: Separation of trait and state in stuttering
Source: Hum Brain Mapp. 2018 Apr 6;39(8):3109–26. doi: 10.1002/hbm.24063 (PMC6055715; doi:10.1002/hbm.24063)
Supplement: Supplementary file 4 — Supporting Information Table II [file HBM-39-3109-s004.docx]

Supplementary Table II: Within-trait tasks effects during scanning in PWS: Regions where there was reduced activity in both subgroups during sentence reading relative to picture description. See the legend to Supplementary Table I for further details.

| Brain region | # voxels | Z statistic | X | Y | Z |
| --- | --- | --- | --- | --- | --- |
| Right medial and orbitofrontal cortex | 2034 |  |  |  |  |
| Right cingulate gyrus (anterior) * |  | 3.89 | 12 | 42 | -2 |
| Right frontal pole |  | 3.73 | 40 | 48 | -6 |
| Left cingulate gyrus (anterior) * |  | 3.38 | -6 | 38 | 4 |
| Left lateral peri-Sylvian cortex | 924 |  |  |  |  |
| Left supramarginal gyrus * |  | 4.03 | -62 | -24 | 24 |
| Left postcentral gyrus * |  | 3.79 | -66 | -22 | 30 |
| Left central operculum |  | 3.56 | -60 | -10 | 8 |
| Right inferior parietal cortex | 2869 |  |  |  |  |
| Right supramarginal gyrus * |  | 4.12 | 58 | -38 | 40 |
| Right postcentral gyrus * |  | 4.03 | 68 | -20 | 24 |
| Posterior medial cortex | 1347 |  |  |  |  |
| Right cingulate gyrus (posterior)* |  | 4.09 | 6 | -40 | 26 |
| Left cingulate gyrus (posterior)* |  | 3.25 | -4 | -40 | 26 |
| Right precuneus |  | 2.97 | 6 | -74 | 32 |
