# Supplementary material for: Separation of trait and state in stuttering
Source: Hum Brain Mapp. 2018 Apr 6;39(8):3109–26. doi: 10.1002/hbm.24063 (PMC6055715; doi:10.1002/hbm.24063)
Supplement: Supplementary file 5 — Supporting Information Table III [file HBM-39-3109-s005.docx]

Supplementary Table III: Task effects within DYS subgroup: Regions where there were differences between picture description and sentence reading tasks. See the legend to Supplementary Table I for further details.

| Brain region | # voxels | Z statistic | X | Y | Z |
| --- | --- | --- | --- | --- | --- |
| Medial and orbitofrontal cortex | 1122 |  |  |  |  |
| Right frontal orbital cortex |  | 4 | 22 | 42 | -8 |
| Right cingulate gyrus (anterior)* |  | 3.9 | 8 | 42 | -2 |
| Left cingulate gyrus (angerior)* |  | 3.44 | -4 | 34 | 0 |
| Left lateral peri-Sylvian cortex | 2334 |  |  |  |  |
| Left central opercular cortex* |  | 4.06 | -50 | -4 | 10 |
| Left superior temporal gyrus |  | 3.99 | -58 | -38 | 8 |
| Right lateral peri-Sylvian and posterior cortex |  |  |  |  |  |
| Right cingulate gyrus (posterior)* | 21263 | 5.35 | 2 | -38 | 26 |
| Right central opercular cortex* |  | 3.93 | 56 | -10 | 14 |
| Right middle temporal gyrus |  | 4.62 | 62 | -26 | -4 |
| Right supramarginal gyrus |  | 5.34 | 54 | -38 | 44 |
| Left cingulate gyrus (posterior)* |  | 4.86 | -2 | -52 | 28 |
| Right lateral occipital cortex |  | 5 | 52 | -58 | 48 |
