# Supplementary material for: Separation of trait and state in stuttering
Source: Hum Brain Mapp. 2018 Apr 6;39(8):3109–26. doi: 10.1002/hbm.24063 (PMC6055715; doi:10.1002/hbm.24063)
Supplement: Supplementary file 6 — Supporting Information [file HBM-39-3109-s006.docx]

**Supplementary Materials: Task effects**

**We included in all models a variable coding for differences between tasks. However, those results are secondary to the design, which was to isolate effects in stuttering. The significant task differences are therefore reported below for each of the models used to isolate trait, subgroup, and state effects in stuttering, respectively, in the current study.**

**Trait analyses**

**Large clusters extended across peaks in lateral occipital and posterior parietal cortices including angular and supramarginal gyri through sensorimotor cortices, including pre and post central gyri, into the insula as well as middle temporal cortex, all occurring bilaterally. In addition, medial activation distinguished these speaking tasks in the posterior parietal and precuneal cortices extending into posterior cingulate cortex and the supplementary motor area, distributed bilaterally. Frontal medial activation, also occurring bilaterally, showed greater activity for the picture description condition than the sentence reading condition extending into paracingulate, and cingulate cortices as well. Notably, task differences in only three peaks were observed to occur unilaterally: right hemisphere lateral frontal pole, right hemisphere middle frontal gyrus extending into superior frontal gyrus, and left hemisphere central opercular cortex extending into posterior insular cortex and posterior superior temporal gyrus (Supplementary Table I, Supplementary Figure 1).**

**Subgroup analyses**

**Specific peaks of increased activity during picture description relative to sentence reading occurred bilaterally in supramarginal gyrus extending into lateral occipital cortex and pre and postcentral gyri, and in the medial structures of the cingulate gyrus (anterior and posterior) and the precuneus (Supplementary Table II).**

**State analyses**

**Task effects were especially pronounced on the medial surface, with greater activity seen for picture description relative to sentence reading in a large cluster extending through posterior cingulate cortex and precuneus into supplementary motor area and postcentral gyrus, bilaterally. The same cluster extended laterally into right hemisphere occipital cortex and supramarginal gyrus, through central opercular cortex into superior and middle temporal gyri and inferior frontal cortex. In the left hemisphere, a large cluster was also observed in central opercular cortex extending into superior temporal gyrus and frontal opercular cortex. The effect of task in this model was therefore consistent those reported above (Supplementary Table III).**
